# Supplementary material for: Changes in hospital staff’ mental health during the Covid‑19 pandemic: Longitudinal results from the international COPE-CORONA study
Source: PLoS One. 2023 Nov 16;18(11):e0285296. doi: 10.1371/journal.pone.0285296 (PMC10653404; doi:10.1371/journal.pone.0285296)
Supplement: S1 Table — (DOCX) [file pone.0285296.s001.docx]

**Supporting information – S1**

**S1 Table.**

| **Country** | **N** | **%** |
| --- | --- | --- |
| Andorra | 89 | 14.5 |
| Germany | 426 | 69.6 |
| Iran | 12 | 2 |
| Ireland | 15 | 2.4 |
| Italy | 42 | 6.9 |
| Romania | 1 | 0.2 |
| Spain | 27 | 4.4 |
