# Supplementary material for: Changes in hospital staff’ mental health during the Covid‑19 pandemic: Longitudinal results from the international COPE-CORONA study
Source: PLoS One. 2023 Nov 16;18(11):e0285296. doi: 10.1371/journal.pone.0285296 (PMC10653404; doi:10.1371/journal.pone.0285296)
Supplement: S2 Table — Notes: PHQ-2 = Patient Health Questionnaire-2; GAD-2 = General Anxiety Disorder-2. (DOCX) [file pone.0285296.s002.docx]

**Supporting information – S2**

**S2 Table.**

| **Variable** | **Total sample**  **(N = 611)** | |  |  |  |
| --- | --- | --- | --- | --- | --- |
|  | **T1**  *Mean (SD)* | **T2**  *Mean (SD)* | **t** | **p** | **d** |
| PHQ-2 | 1.52 (1.27) | 1.79 (1.32) | 5.15 | <.001 | 0.21 |
| GAD-2 | 1.46 (1.39) | 1.60 (1.36) | 2.61 | .009 | 0.25 |

*Notes*: PHQ-2 = Patient Health Questionnaire-2; GAD-2 = General Anxiety Disorder-2
