# Supplementary material for: Changes in hospital staff’ mental health during the Covid‑19 pandemic: Longitudinal results from the international COPE-CORONA study
Source: PLoS One. 2023 Nov 16;18(11):e0285296. doi: 10.1371/journal.pone.0285296 (PMC10653404; doi:10.1371/journal.pone.0285296)
Supplement: S3 Table — Notes: PHQ-2 = Patient Health Questionnaire– 2; GAD-2 = General Anxiety Disorder-2. All values are standardized regression weights. *p < .05; **p < .01; ***p < .001. (DOCX) [file pone.0285296.s003.docx]

**Supporting information – S3**

**S3 Table.**

|  |  |  | **Time** | | **Time*Group** | |
| --- | --- | --- | --- | --- | --- | --- |
| **Variables, mean (SD)** | **A**  **Nuremberg**  **N = 134** | **B**  **Other centers**  **N = 243** | **F** | **η^2^** | **F** | **η^2^** |
| PHQ-2 |  |  |  |  |  |  |
| T1 | 0.78 (0.63) | 0.70 (0.65) | 26.39*** | .41 | 1.23 | .002 |
| T2 | .90 (0.64) | .88 (0.71) |  |  |  |  |
| GAD-2 |  |  |  |  |  |  |
| T1 | 0.65 (0.65) | 0.91 (0.76) | 3.21 | .005 | 1.76 | .003 |
| T2 | 0.74 (0.65) | 0.93 (0.72) |  |  |  |  |

*Notes*: PHQ-2 = Patient Health Questionnaire – 2; GAD-2 = General Anxiety Disorder-2.

All values are standardized regression weights. *p < .05; **p < .01; ***p < .001.
